# Transition of differential histone H3 methylation in photoreceptors and other retinal cells during retinal differentiation

**DOI:** 10.1038/srep29264

**Published:** 2016-07-05

**Authors:** Kazuko Ueno, Toshiro Iwagawa, Hiroshi Kuribayashi, Yukihiro Baba, Hiromitsu Nakauchi, Akira Murakami, Masao Nagasaki, Yutaka Suzuki, Sumiko Watanabe

**Affiliations:** 1 Division of Molecular and Developmental Biology, Institute of Medical Science, University of Tokyo, Tokyo, Japan; 2 Division of Biomedical Information Analysis, Department of Integrative Genomics, Tohoku Medical Megabank Organization, Tohoku University, Sendai, Miyagi, Japan; 3 Division of Stem Cell Therapy, Center for Stem Cell Biology and Regenerative Medicine, Institute of Medical Science, University of Tokyo, Tokyo, Japan; 4 Department of Ophthalmology, Graduate School of Medicine, Juntendo University, Tokyo, Japan; 5 Department of Medical Genome Sciences, Graduate School of Frontier Sciences, University of Tokyo, Chiba, Japan

## Abstract

To analyze cell lineage-specific transitions in global transcriptional and epigenetic changes during retinogenesis, we purified retinal cells from normal mice during postnatal development into two fractions, namely, photoreceptors and other retinal cells, based on Cd73 expression, and performed RNA sequencing and ChIP sequencing of H3K27me3 and H3K4me3. Genes expressed in the photoreceptor lineage were marked with H3K4me3 in the Cd73-positive cell fraction; however, the level of H3K27me3 was very low in both Cd73-positive and -negative populations. H3K27me3 may be involved in spatio-temporal onset of a subset of bipolar-related genes. Subsets of genes expressed in amacrine and retinal ganglion cells, which are early-born retinal cell types, were suggested to be maintained in a silent state by H3K27me3 during late-stage retinogenesis. In the outer nuclear layer, upregulation of Rho and rod-related genes were observed in Ezh2-ablated retina, suggesting a role for H3K27me3 in the maintenance of proper expression levels. Taken together, our data on the transition of lineage-specific molecular signatures during development suggest that histone methylation is involved in retinal differentiation and maintenance through cell lineage-specific mechanisms.

The vertebrate neural retina is organized into a laminar structure comprised of six types of neurons and glial cells, Müller glia, and astrocytes. Rod photoreceptors form the outer nuclear layer (ONL) together with the cone. In the inner nuclear layer (INL), interneurons such as horizontal, amacrine, and bipolar cells are found in addition to the Müller glia cell body. In the mouse, these major retinal cell classes are generated from a common population of multipotent retinal progenitor cells between embryonic day (E) 11 and postnatal day (P) 10 in a conserved temporal order^1^. Retinal ganglion cells (RGCs), amacrine cells, cone photoreceptors, and horizontal cells differentiate at relatively early stages, primarily before birth, while bipolar cells and rod photoreceptors are mainly generated at later stages after birth^2^,^3^.

As in other tissues, epigenetic mechanisms play pivotal roles in retinal health and diseases. DNA methylation had been well-documented in retinal development and is involved in diseases such as diabetic retinopathy and retinoblastoma^4^,^5^. Methylation of basic amino acids in histone proteins is a crucial epigenetic mechanism for gene expression regulation^6^. Failed histone methylation is a cause of various diseases, including cancer. Retinal development is also regulated by various histone modifications. The dynamics of various histone markers in chromatin organization of mouse rod photoreceptors during development has been reported^7^. Ehmt2/Kmt1c/G9a, a histone H3K9/K27 demethylase, is essential for the differentiation and survival of retinal cells^8^. Nrl ChIP sequence revealed that Nrl bound to Kdm5b gene, a histone H3K4 demethylase, and loss of function analysis supported the importance of Kdm5b in rod maintenance^9^.

The di- and tri-methylation of lysine 27 on histone H3 (H3K27me2/3) by Ezh2/Kmt6 together with polycomb repressive complex 2 (PRC2) is a gene repression mechanism^10^,^11^,^12^. We focused on the roles of H3K27me3 during retinal development. Based on loss of function analyses of Ezh2 and Jmjd3, we reported that H3K27me3 modification in Bhlhb4 and Vsx2 genes is critical for the maturation of PKC- and Recoverin-positive bipolar cell subsets^13^,^14^. INL-specific expression of Jmjd3 plays a role in the cell lineage specific function of H3K27me3. Interestingly, examination of the detailed expression pattern of Ezh2 and Jmjd3 during retinal development also showed striking differential expression patterns of Ezh2 between the ONL and INL during retinal development, suggestive of the differential roles of the H3K27 methylation system in cell lineages in the ONL and INL. H3K4 methylation is one of the most well-studied histone methylation patterns, and it acts positively on the transcription of target genes^6^. Previous studies examining the comprehensive genomic map using chromatin immunoprecipitation sequencing (ChIPseq) showed that genes expressed in mature rod photoreceptors have a unique signature of *de novo* H3K4me2 accumulation^15^.

Therefore, it is important to analyze histone modifications in a cell type-specific manner, especially in segregating rod and other cell types. In mouse, rod commitment occurs on embryonic day 12 (E12) to postnatal day 7–8 (P7–8), and differentiation peaks after birth^2^,^3^. We purified rod and photoreceptor precursors using the specific expression of Cd73^16^ from mouse retina at P2, P5, and P8. From the same retinal samples, we also purified a Cd73-negative population consisting of interneurons, Müller glia, and RGCs. RNA sequencing (RNAseq) and ChIPseq for H3K4me3 and H3K27me3 were performed. We found that cell lineage- and genes-specific modifications of histones during retinal development.

## Results

### The outer nuclear layer (ONL) and inner nuclear layers (INL) are differentially affected by the loss of histone H3K27 methylation in Ezh2 knockout mice during retinal development

**Figure 1:**
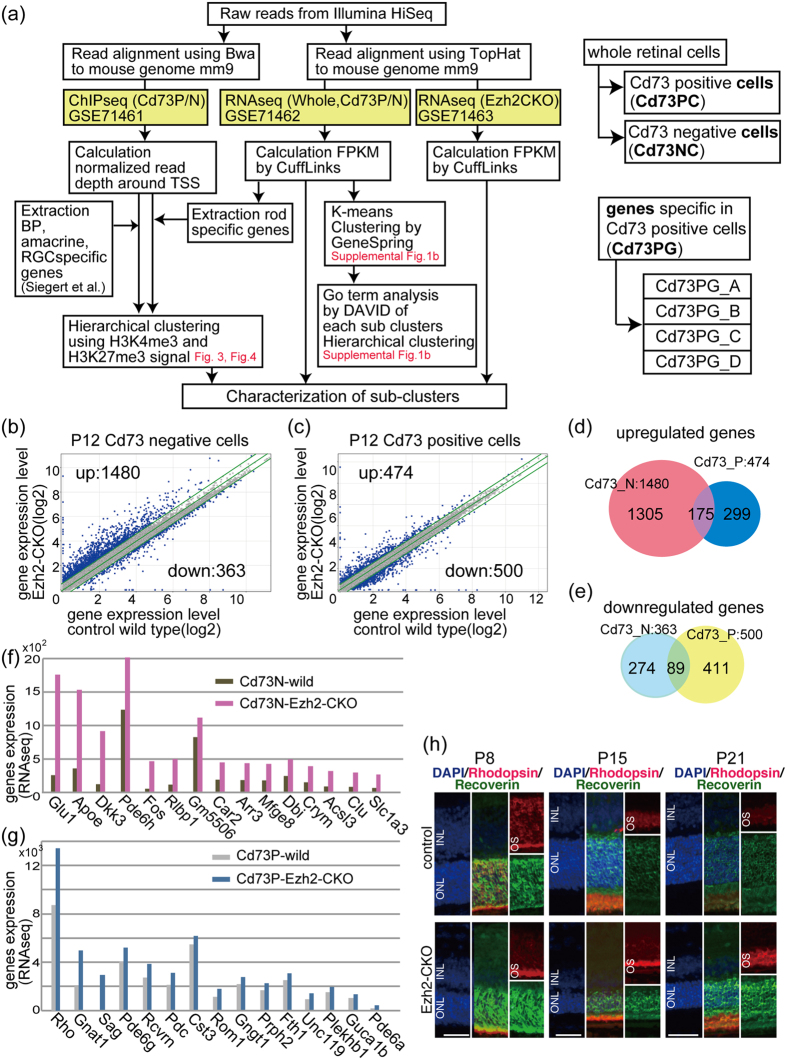
Modulation of Ezh2 or Jmjd3 resulted in reduced thickness of INL and ONL. (**a**) Schematic diagram of the flow of analyses. Right panels show definition of the cells and genes. (**b,c**) Gene expression levels of Cd73 negative (**b**) or Cd73 positive (**c**) populations of wild control (horizontal axis) and Ezh2-CKO (vertical axis) are shown. Diagonal lines indicate median (middle line) and +/−1.5 fold difference values (upper and lower lines) in the absolute value. Numbers of genes with more or less than 1.5 times of expression level in Ezh2-CKO retina than control were indicated as up or down, respectively, and are shown in the corner of panels. (**g,h**). Venn diagram showing overlapping of genes of up (**d**) or down (**e**) regulated genes in Ezh2-CKO retina. (**f,g**) Top 15 genes of upregulated in Cd73 negative cells (**f**) or Cd73 positive cells (**g**) of Ezh2-CKO retina. (**h**) Immunostaining patterns of Rhodopsin and Recoverin of Ezh2-CKO or control retina at indicated developmental stages. Nuclei were stained with DAPI. INL, inner nuclear layer; ONL, outer nuclear layer; OS, outer segment. Scale bars = 50 μm (**h**).

We previously examined the phenotype of retina-specific Ezh2 conditional knockout mice (Ezh2-CKO) and found that the thickness of Ezh2-CKO retina was comparable to control retina during the embryonic period but decreased slightly after birth; the reduction became prominent at around P6^14^. To examine the molecular signatures of ONL and other cells separately in this mouse model, we performed flow cytometry using Cd73, which is specifically expressed in precursor and mature rod photoreceptors^16^. Cd73 expression was observed from E18 by RT-PCR, and Cd73 protein was detectable from P1 by flow cytometry^16^. We performed RNAseq using purified Cd73-positive (P)- and CD73-negative (N)-populations of Ezh2-CKO retina and processed the obtained sequence data as shown in Fig. 1a. We used P12 mouse retinas because at this stage, photoreceptors begin to degenerate in the mutant retina, and the expression of gene subsets in bipolar cells and photoreceptors, both of which differentiate during later stages of retinal development, is apparent.

Global changes in gene expression in Ezh2-CKO showed a large number of upregulated genes in the Cd73N population (Fig. 1b). However, fewer genes were upregulated in the Cd73P population (Fig. 1c), which is in accordance with the low expression level of Ezh2 in the Cd73P population^13^. Only some genes were commonly upregulated (Fig. 1d) or downregulated (Fig. 1e) between the Cd73P and Cd73N populations. We listed the top 15 genes upregulated in the Cd73N (Fig. 1f) and Cd73P (Fig. 1g) fractions, and Müller glia-related genes, such as *Apoe, Dkk3, Rlbp1, Dbi,* and *Slc1a3*, were enriched in the Cd73N list. The majority of the genes in the Cd73P list were known rod-specific genes. We next examined protein expression of Rho and Rcvrn, which were upregulated in the Cd73P population in Ezh2-CKO retina, by immunostaining. Rhodopsin and Rcvrn showed stronger signals in Ezh2-CKO retina than in control retina at all examined developmental stages (Fig. 1h). Since previous studies indicated that overexpression of Rho leads to photoreceptor degeneration^17^,^18^, we hypothesized that elevated expression of Rho may play a role in the degeneration of ONL in Ezh2-CKO. Taken together, different roles were thought to be involved in the reduction of INL and ONL in Ezh2-CKO, supporting the importance of independent histone methylation analysis of INL and ONL.

### Comprehensive analysis of gene expression patterns in rod photoreceptors and other cells during retinal development, using Cd73-based cell fractionation, and classification of genes according to their expression patterns

To examine the molecular signature of ONL and other cells independently during the postnatal period of mouse retina, which is a critical period for late-born retinal subset development, we purified Cd73P and Cd73N cell fractions from mouse retina at P2, P5, and P8; the purified cell fractions were designated as Cd73 positive cells (Cd73PC) and Cd73 negative cells (Cd73NC) (Fig. 1a). We also prepared whole retinas at E15 and E18, which consisted of mostly progenitor cells and early-born retinal subtypes such as retinal ganglion cells (RGCs), cone, and horizontal cells. These cell fractions were then subjected to RNAseq to obtain a comprehensive gene expression pattern. To verify the RNAseq results for analysis of cell lineage molecular signatures, we categorized the list of genes (19,954 genes) into 15 distinct clusters using K-means according to their expression patterns obtained using GeneSpring (Agilent Technologies) (Supplemental Fig. 1a). Approximately 7,000 genes were not expressed in any stage or subset of the retina (Supplemental Fig. 1a, C2 cluster), and we analyzed all clusters (excluding C2) based on gene ontology (GO) analysis. GO terms with <0.25 Benjamini values were chosen, and hierarchical clustering using -log10 p-values by DAVID analysis^19^ identified roughly five major groups (Supplemental Fig. 1b). Genes in group 1 (C1, C3) were expressed highly in retinal progenitors and decreased rapidly in Cd73P photoreceptors after birth (Supplemental Fig. 1a). These genes were mostly correlated with cell cycle and proliferation (Supplemental Table 1, C1, and C3), suggesting that cell cycle-related genes disappear rapidly when cells are committed to rod photoreceptor differentiation. The GO terms of group 2 (C5, C12) were related to translation and metabolic pathways (Supplemental Table S1, C5, C12), suggesting that genes involving basic physiological activities were maintained at high expression levels regardless of the developmental stage or cell type. Group 5 genes (C7, C13) were characterized by high expression in rod photoreceptors; a number of light- and vision-related GO terms were found in this group (Supplemental Table S1, C7, and C13).

### Genes specifically expressed in rod photoreceptors were classified according to their expression pattern

**Figure 2:**
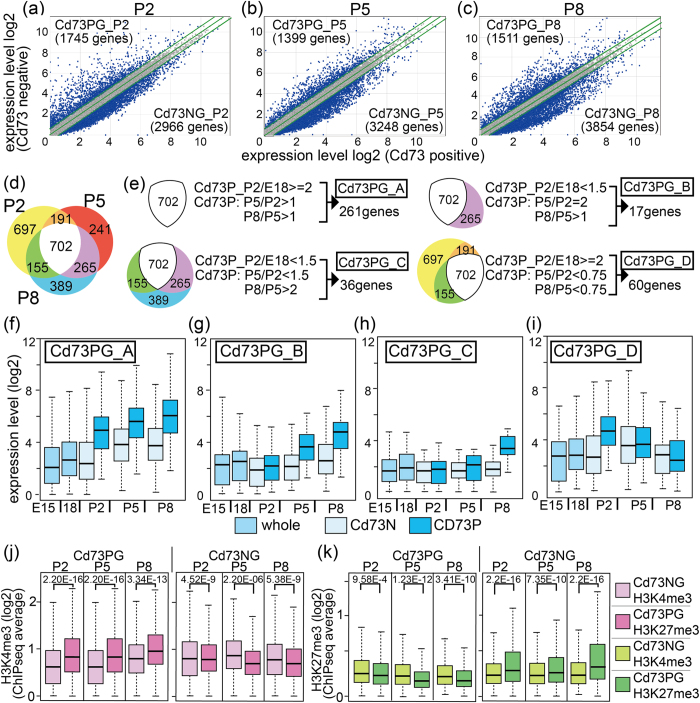
Comparison of RNAseq data of Cd73 positive and negative cell fraction from developing retina. RNAseq of whole retina at E15 and E18, or purified cell fractions of Cd73 positive (Cd73PC) and Cd73 negative (Cd73NC) at P2, P5, and P8 was done. (**a–c**) Global gene expression levels of Cd73PC (horizontal axis) and Cd73NC (vertical axis) patterns at P2 (**a**), P5 (**b**), and P8 (**c**). Diagonal lines indicate median (middle line) and +/−1.5 fold difference values (upper and lower lines). Genes predominantly expressed in either fraction was designated as Cd73P genes (Cd73PG) and Cd73N genes (Cd73NG). The genes were considered Cd73PG or Cd73NG only if expressed at >1.5 fold than opponent fraction. (**d**) Overlapping of upregulated genes in each stage are shown as Venn diagram. (**e**) Genes were grouped by their expression patterns, and classified groups were designated as a to d. (**f–i**) Box plot of average expression level of genes categorized to groups Cd73PG_A to _D. **(j,k**) ChIPseq for H3K4me3 or H3K27me3 was done using Cd73PC or Cd73NC fraction of retina at P2, P5, and P8. Average values of H3K4me3 (**j**) or H3K27me3 (**k**) signals of +/−5kb of TSS in each gene loci of genes in Cd73PG or Cd73NG are shown. Statistical significance values calculated by Kolmogorov-Smirnov test are shown in the top of each panel in (**j,k**).

We next focused on genes specifically expressed in rod photoreceptors and directly compared genes between Cd73P and Cd73N populations at each stage (Fig. 2a–c). First, we examined the expression patterns of several genes reported to belong to certain retinal cell-specific gene subsets and found differential expression of these genes between the cell fractions (Supplemental Fig. 2a). We then chose the genes that were expressed more highly in Cd73PC than Cd73NC or *vice versa*, with 1.5-fold changes in the absolute values at each stage designated as upregulated or downregulated (Fig. 2a–c). Overlap of genes specific in Cd73 positive cells (Cd73PG) at P2, P5, and P8 was examined (Fig. 2d), and the genes were categorized into four groups according to their expression patterns (Fig. 2e). The first group (Cd73PG_A) contained genes expressed specifically in Cd73PC from P2, maintained at high expression levels in Cd73PC at P5 and P8 (Fig. 2e,f). The second group contained genes expressed from P5 in Cd73PC (Cd73PG_B, Fig. 2e,g), and the third contained genes that were expressed in Cd73C at P8 (Cd73PG_C, Fig. 2e,h). Genes expressed in Cd73PC at P2 but that then decreased in the following developmental stages were categorized as group D (Cd73PG_D, Fig. 2e,i).

GO analysis indicated that group A genes were highly enriched in vision functions (Supplemental Table S2A). In classes B, C, and D, there were GO terms unrelated to vision functions, and no terms showed significantly low Benjamini values (Supplemental Table S2B–D), suggesting that the roles of these genes in retinal development have not been shown previously.

Then, we verified the expression of the Cd73PG_A genes with low fold changes, i.e., less than a 2.0-fold difference between Cd73PC and Cd73NC, in the RNAseq data. There were 12 genes with less than a 2.0-fold difference at two or all three of the stages. We examined the expression patterns of these genes by qPCR in newly prepared triplicate samples of Cd73P and Cd73N cell fractions at P2, P5, and P8. In addition, Crx, which is a critical photoreceptor-specific gene and showed less than a 2.0-fold change at P5, was included for validation. All cases, except for one, showed statistically significant differential expression between Cd73P and Cd73N cells (Supplemental Fig. 2b–d), justifying the use of a 1.5-fold threshold.

### ChIP sequencing (ChIPseq) of histone H3K4 and H3K27 trimethylation using Cd73P and Cd73N cell fractions revealed high H3K4me3 and low H3K27me3 levels at photoreceptor-specific gene loci

To analyze the involvement of histone methylation in lineage- and stage-specific gene expression patterns of the retina, we assessed H3K4me3 and H3K27me3 genomic occupancy by ChIPseq using the Cd73PC and Cd73NC fractions from P2, P5, and P8 retina. We first examined the average levels of H3K4me3 and H3K27me3 in Cd73PG and Cd73NG (Fig. 2j,k). Average cumulative signals of H3K4me3 and H3K27me3 at +/−5 kb of the transcription start site (TSS) of Cd73PG or Cd73NG at each stage (Fig. 2a–c) were calculated and shown as a box plot (Fig. 2j,k). The H3K4me3 level of Cd73PG was significantly higher in Cd73PC than Cd73NC (Fig. 2j), suggestive of a positive correlation between the H3K4me3 level and gene expression level. The H3K27me3 level in Cd73PG loci was low in all samples and slightly lower in Cd73NC than in Cd73PC (Fig. 2k, left panel). In contrast, H3K27me3 in Cd73NG showed higher levels in Cd73PC than in the Cd73N population (Fig. 2k, right panel), suggesting that Cd73N-specific genes are suppressed in the Cd73P population by H3K27me3 markers.

To examine the relationship between the transition in expression of photoreceptor-related genes and histone modifications, we directly compared the gene expression levels and H3K4me3 or H3K27me3 modification levels of Cd73PG_A genes (Supplemental Fig. 3a,b). Some genes including *Nrl* and *Crx* contained strong H3K4me3 signals in a Cd73PC-specific manner (Supplemental Fig. 3a inset, 3b), and genome browser trackers confirmed Cd73PC-specific strong H3K4me3 signals around the TSS of these genes (Supplemental Fig. 3d). H3K27me3 level was significantly lower in Cd73PG_A loci both in Cd73PC and Cd73NC (Supplemental Fig. 3a), with a few exceptions (Supplemental Fig. 3a, insets). Strong signals were observed at each gene locus in both Cd73PC and Cd73NC (Supplemental Fig. 3c), but the signal patterns were slightly different (Supplemental Fig. 3d), suggesting that different machinery incorporates H3K27me3 at the same gene locus in different cell lineages.

### Positive correlation of H3K4me3 levels with the expression levels of photoreceptor-specific genes in the Cd73P cell fraction

**Figure 3:**
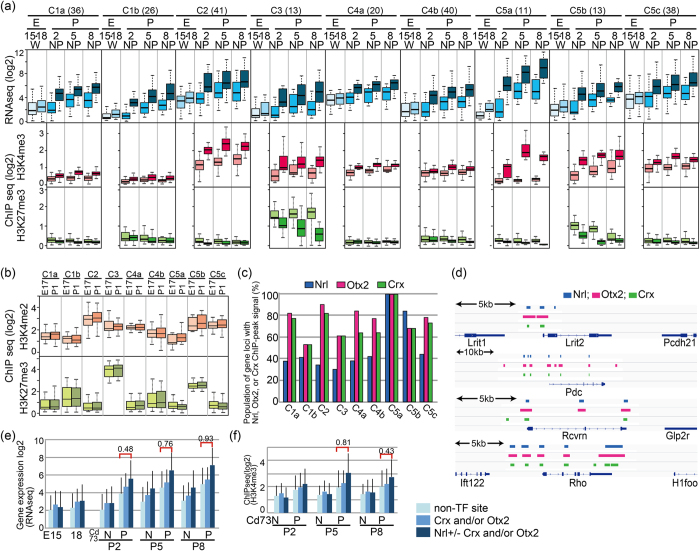
Histone H3K4me3 and H3K27me3 modification of retinal gene loci of Cd73P specific genes. (**a**) Genes of Cd73P were sub-clustered to C1a to C5c, and average levels of gene expression (RNAseq), H3K4me3 (ChIPseq), and H3K27me3 (ChIPseq) of each sub-cluster are shown. E, embryo; P, postnatal; W, whole retinal cells; N, Cd73 negative cells; P, Cd73 positive cells. The digits under E or P indicate days after fertilization or birth, respectively. (**b**) H3K4me2 and H3K27me3 levels of genes in sub-clusters at E17 and P1 whole retina were calculated from data of Popova *et al*.^15^, and average values are shown. (**c**) Presence of ChIP peak signals of Nrl^9^, Crx^20^, or Otx2^21^ was examined for 5 kb +/− region of TSS in gene loci of the sub-clusters. Population (%) of genes having ChIP peak signals in each sub-clusters is shown. (**d**) Genomic browser tracks of Nrl, Otx2, and Crx binding peaks in representative Cd73P-genes. (**e,f**) Average gene expression level (**e**) and H3K4me3 level (**f**) of genes having ChIP peaks of Nrl, Otx2, or Crx in their genomic loci are shown. p values were calculated by student’s T test, and effect size of the results was examined by Pearson’s correlation coefficient (r).

To examine the roles of H3K4me3 and H3K27me3 gene expression in more detail, we performed hierarchical clustering of Cd73PG_A by using values of histone H3K4me3 and H3K27me3 of Cd73PC and Cd73NC at P2, P5, and P8. We found that there were roughly nine different sub-clusters in Cd73PG_A (Supplemental Fig. 4a). Although we did not consider expression levels for clustering, genes in the same sub-clusters showed strikingly similar expression patterns (Fig. 3a), suggestive of a contribution of H3K4me3 and H3K27me3 in gene expression patterns. H3K4me3 levels in some sub-groups were very low, suggesting that H3K4me3 modification contributes to the high expression of photoreceptor-related genes, but only in limited cases.

We then explored H3K4 and H3K27 methylation levels in embryonic retina using published H3K4me2 and H3K27me3 ChIPseq data from E17 and P1 whole retina^15^. Interestingly, levels of H3K4me2 and H3K27me3 in E18 and P1 retina showed good correlations with those of P2 retina (Fig. 3b), suggesting that H3K27 methylation had already occurred at a certain subset of gene loci in progenitor cells.

### Ingenuity pathways analysis (IPA)-predicted the role of Nrl in Cd73P gene expression

To explore the involvement of common upstream regulators of genes in the subclasses, we performed IPA analysis. The IPA results revealed several transcription factors with significant p-values, and it was striking that 6 out of 11 genes within the C5a cluster were thought to be downstream of Crx (Supplemental Table S3). Nrl was also thought to be upstream of several genes in C1b, C5a, and C5c (Supplemental Table S3). We then analyzed potential binding of these transcription factors at the gene loci using public available ChIPseq data for Nrl^9^, Crx^20^, and Otx2^21^. Gene populations containing ChIPseq signals in each cluster showed that all genes classified in C5a contained Nrl peak signals (Fig. 3c). The majority of genes with Nrl ChIPseq peaks were also associated with Otx2 and Crx ChIPseq peaks, and these sites were located at similar positions (Fig. 3d). The expression level of genes with Nrl peaks was significantly higher than those of genes without Nrl peaks in Cd73PC (Fig. 3e), and similar tendencies were observed for the H3K4me3 level (Fig. 3f), which confirmed the contribution of Nrl to photoreceptor-related gene expression via H3K4me3 modification. In contrast, expression levels of genes with Crx and/or Otx2 peaks (but not Nrl peaks) at their loci were indistinguishable from those without the peaks (Fig. 3e).

### The gene loci of upregulated photoreceptor-related genes and Cdk2a in Ezh2-CKO retina contained weak H3K27me3 signals

As shown in Fig. 1g, the number of photoreceptor-related genes was upregulated in Cd73P cells from Ezh2-KO retina. The 18 Cd73P_A genes were upregulated in Ezh2-CKO, and genome browser snapshots showed broad and weak H3K27me3 signals in the genomic regions surrounding these genes (Supplemental Fig. 5a). Previously, H3K27me3 enrichment was reported along the entire gene locus of Ink4a-Arf (Cdk2a), acting as a protective blanket to maintain repression of the target gene^22^. Cdk2a was de-suppressed in the Cd73NC, but not Cd73PC, cell population (Supplemental Fig. 5b), and we found a blanket-type pattern in the H3K27me3-modified regions of the Cdk2a locus in the Cd73NC, but not Cd73PC, cell population (Supplemental Fig. 5c).

### Histone modifications associated with genes expressed in bipolar, amacrine, and RGCs differed from those of photoreceptor-specific genes

**Figure 4:**
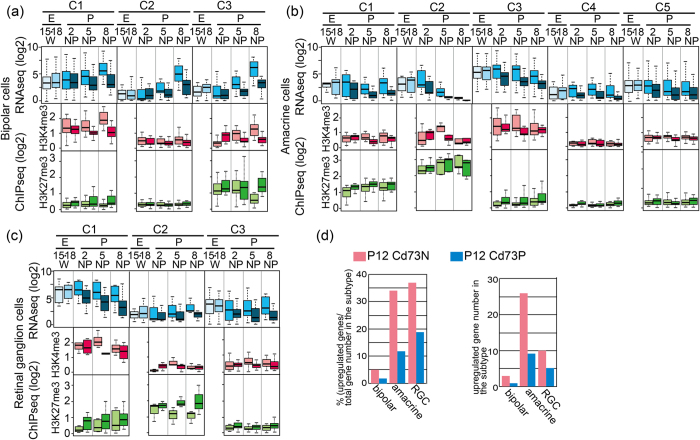
Analysis of RNAseq, ChIPseq data of bipolar, amacrine, and retinal ganglion cell specific genes. (**a–c**) Genes of bipolar (**a**), amacrine (**b**), or retinal ganglion cell (RGC, **c**) were sub-clustered (Supplemental Fig. 4), and average levels of gene expression (RNAseq), H3K4me3 (ChIPseq), and H3K27me3 (ChIPseq) of each sub-cluster are shown. E, embryo; P, postnatal; W, whole retinal cells; N, Cd73 negative cells; P, Cd73 positive cells. The digits under E or P indicate days after fertilization or birth, respectively. (**d**) Population and number of upregulated genes in Ezh2-CKO of bipolar, amacrine, RGC specific genes is shown. Genes are considered as upregulated only if expression is 1.5 fold higher in Ezh2-CKO than control.

We next examined genes regulating retinal interneurons and RGCs. To accomplish this, we used previously published information on cell type-specific genes^23^. We first examined expression patterns of genes categorized as bipolar-, amacrine-, or RGC-specific in a previous report^23^ using our RNA sequence data of Cd73PC and Cd73NC. To explore the Cd73N-specific expression of genes, we calculated relative expression levels of each gene in Cd73NC to that in Cd73PC, and clustered genes according to their expression pattern (Supplemental Fig. 4b,c). A cluster of genes that did not show higher expression in Cd73PC than in Cd73NC were excluded from further analyses (Supplemental Fig. 4b,c, grey colored clusters). Remaining genes in each subclass were designated as BipolarG, AmacrineG, and RGCG. We then compared expression levels versus H3K4me3 or H3K27me3 levels for these genes (Supplemental Fig. 6a–c). Gene expression and H3K4me3 levels showed a positive relationship in most cases in Cd73NC. Levels of H3K27me3 were scattered and had weak or no correlation with gene expression levels (Supplemental Fig. 6). Hierarchical clustering of genes according to H3K4me3 and H3K27me3 levels yielded sub-clusters (Supplemental Fig. 4e–g), and again (as in the case of Cd73PG_A, although we did not include information on expression levels for the clustering) sub-clusters showed their specific characteristic gene expression patterns (Fig. 4a–c). A variety of H3K27me3 patterns were observed among subclusters.

### BipolarG genes may include targets of the H3K27me3 demethylase Jmjd3

**Figure 5:**
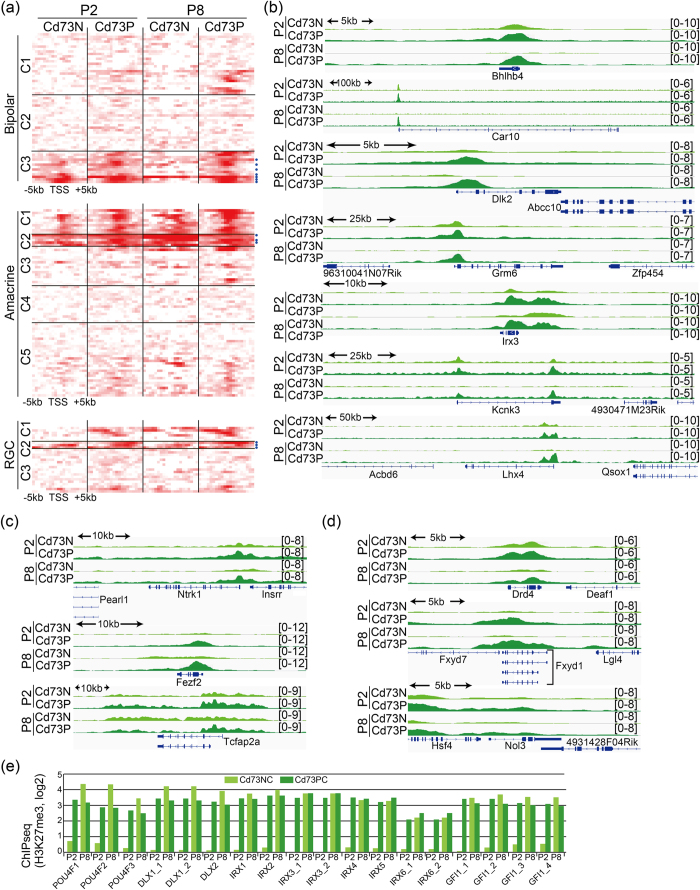
Genome browser tracks of H3K27me3 signals in gene loci of bipolar, amacrine, RGC. (**a**) Heat map of H3K27me3 occupancy for 10 kb region around TSS. Each row indicates H3K27me3-modified DNA fragment. Colors indicate enrichment for H3K27me3. Order of genes is the same order with that in Supplemental Fig. 4. (**e–g**) Genome browser snapshots showing the indicated region of log2 ratio enrichment for H3K27me3. Genes in C3 subclass of bipolar (**b**), C2 subclass of amacrine (**c**), and C2 subclass of RGC (**d**) are shown.

H3K27me3 levels of BipolarG_C3 were high, excluding Cd73NC in the P8 population (Fig. 4a), and C3 contained *Bhlhb4*, which was previously identified as a target of Jmjd3, suggesting that these genes are de-methylated by Jmjd3, which was expressed specifically in postnatal INL^13^. A heat map of H3K27me3 tracks around the TSS of each gene showed that the number of genes in bipolar C3 have strong modifications in both Cd73PC and Cd73NC fractions at P2 around TSS and disappeared in Cd73NC at P8 corresponding to the stage of bipolar cell differentiation (Fig. 5a), suggesting these genes are additional direct targets of Jmjd3. Genome browser patterns clearly indicated a loss of H3K27me3 peaks around TSS in bipolar C3-related genes in the Cd73NC fraction at P8 (Fig. 5b).

### Genes related to early-born amacrine and RGC subsets showed high H3K27me3 levels

AmacrineG_C2 showed high levels of H3K27me3 in all samples (especially P5 and P8), which may contribute to the low expression level of the genes in P5 and P8 (Figs 4b and 5a,c). Since amacrine is an early developing subtype, genes were likely turned off after differentiation was complete. Similarly, RGCG_C1 showed high H3K4me3 levels in both Cd73NC and Cd73PC, but high levels of K27me3 in Cd73PC, probably to suppress the expression of these genes in Cd73PC (Fig. 4c). RGCG_C2 genes had conspicuously high H3K27me3 levels in Cd73P, highlighting the importance of turning these genes off in Cd73P cells (Fig. 4c). In addition, H3K27me3 levels in Cd73NC were low for RGCG_C2 genes compared with those in Cd73PC (Figs 4c and 5a,d), suggesting that maintaining high expression levels of these genes is important for RGC functioning. This observation is in agreement with the high expression levels of Jmjd3 in RGC^13^. More than 30% of genes categorized as amacrine- and RCG-specific were upregulated in both the Cd73PC and Cd73NC fractions of Ezh2-CKO retina (Fig. 4d, Table 1).

Several transcription factors are expressed almost exclusively in RGCs, such as Dlx1/2, Pou4f, Irx, and Gfl1 family members^24^,^25^,^26^. We examined the H3K27me3 modifications associated with these gene loci; surprisingly, very weak signals were seen in the Cd73NC fraction, but very strong signals were seen in the Cd73PC fraction at P2, as well as in both fractions at P8 (Fig. 5e). However, the expression levels were not augmented in Ezh2-KO retina, suggesting that methylases other than Ezh2, probably Ezh1, are responsible for the high H3K27me3 levels at these gene loci.

## Discussion

In this report, we analyzed the changes in the molecular signatures of Cd73P photoreceptors and other retinal cells that were Cd73N cells during a critical differentiation period in retina. The data include gene expression and histone modification patterns in these cell lineages at different developmental time points. Such multi-dimensional datasets of primary tissue can be used to analyze cell lineage-specific molecular events during differentiation. Previous studies using photoreceptor-specific knockout mice revealed many molecular aspects that occur specifically in photoreceptor lineages^27^. Our data further revealed and highlighted the dynamics of photoreceptors, as well as other retinal neuron-specific events by direct comparison of molecular events in photoreceptors and other cells. This is a powerful approach to analyze cell lineage-specific molecular events; however, we need to be careful of the risk of artifacts introduced by cell sorting, although we performed cell sorting quickly at the expense of purity. For lineage-specific analysis of the Cd73N population, which is a mixture of a few cell lineages, we need to isolate the individual cell lineages, but so far the appropriate cell surface antigens have not been identified. However, we previously used an anti-Isl1 antibody to purify cells for ChIP qPCR application, and we are currently investigating applicable antibodies for use in isolating other cell lineages for this purpose.

Previous studies observed strong H3K4me2 signals at the loci of rod-specific genes^15^. We found that photoreceptor-specific gene loci were also marked by H3K4me3; however, this was not observed in non-photoreceptor cells. Based on clustering according to histone modification patterns, we observed various patterns of H3K4me3 modification in each sub-cluster, suggesting that rod-specific genes are not regulated uniformly but rather through multiple mechanisms. In most cases, H3K4me3 signals were already observed at the P2 stage, suggesting that H3K4me3 is involved in the initial stage of transcription. We explored whether this photoreceptor lineage-specific H3K4me3 signal was gained after commitment to the photoreceptor lineage, and ChIPseq data of the whole retina at E17 and P1 by Popova *et al*. indicated that levels of H3K27me3 in progenitors were similar to levels of Cd73P at P2^15^. These finding suggested that photoreceptor-specific H3K4me3 signals were present even before apparent differentiation into photoreceptors. However, determining the level of selectivity to the photoreceptor-related gene loci in retinal progenitor cells requires further study. IPA analysis revealed strong involvement of Nrl, Crx, and Otx2, which are well-documented as critical transcription factors for photoreceptor development^27^, as upstream molecules in certain subclasses of genes. Nrl ChIPseq identified the Kdm5b gene locus as one of the binding sites of Nrl^9^, and our data suggested a contribution of Nrl to photoreceptor-specific gene expression levels, as well as H3K4me3. However, we could not identify specific Nrl binding sites in more than half of Cd73P-A gene loci, suggestive of involvement of other transcription factor(s) for H3K4me3 modification during the very early stages of photoreceptor differentiation.

H3K27me3 modification of a subset of bipolar-related genes was downregulated in the Cd73N fraction at P8, which is a stage corresponding to bipolar cell maturation; this result is consistent with our previous report that Jmjd3 plays a pivotal role in the de-methylation of bipolar-related genes to achieve temporally spatially appropriate expression. Our previous study suggested that *Bhlhb4* and *Vsx1* are direct target gene loci of the Ezh2/Jmjd3 system^13^, and current studies revealed additional genes categorized as bipolar that are potential direct targets of Ezh2/Jmjd3. We observed strong Cd73P fraction-specific H3K27me3 signals in several genes of not only bipolar, but also amacrine cells and RGC. This suggests that H3K27me3 is involved in a mechanism to protect and maintain rod photoreceptors by preventing unnecessary or toxic gene expression for their differentiation or maintenance.

As a potential mechanism for ONL degeneration in Ezh2-KO retina, we observed upregulation of rhodopsin. Excess rhodopsin was reported to lead to photoreceptor degeneration in transgenic mouse^17^,^18^, supporting our hypothesis. However, investigation of H3K27me3 signals at the Rho locus showed broad, but weak, H3K27me3 signals along the entire locus, suggesting that regulation of Rho and other genes by H3K27me3 may differ from that of bipolar-related genes, which display H3K27me3 signals around the TSS. A previous report suggested that broad enrichment across gene bodies corresponds to transcriptional inhibition^28^, but since the H3K27me3 signals were weak, we also need to consider the indirect effects of Ezh2-KO on Rho upregulation.

We previously showed that Ezh2 was strongly expressed in retinal progenitor cells, and after differentiation, it was expressed exclusively in RGC and INL at the protein level^13^. There are two homologs in mammalian Ezh: *Ezh1* and *Ezh2*^29^. As reported in other tissues^30^, *Ezh1* was only very weakly expressed in proliferating cells in the retina, but was weakly expressed in both Cd73P and N fractions at later stages. Although immunostaining did not detect expression of Ezh2 in ONL^13^, we observed weak *Ezh2* signals in Cd73PC by RNAseq, suggestive of a contribution of Ezh2 in ONL. Previous studies proposed a model suggesting the importance of PRC2-Ezh2 for *de novo* establishment of H3K27me3 in dividing cells, whereas PRC2-Ezh1 is required for its maintenance in resting cells^31^. Changing the level of another member of the PRC2 complex, Jarid2, plays important roles for switching from Ezh2 to Ezh1 involved in the PRC2 complex^31^. Expression of *Jarid2* in our RNAseq data was indicative of strong and ubiquitous expression of *Jarid2* in retina, suggesting that Jarid2 may not be a key factor responsible for the switch between Ezh2 and Ezh1 expression, and further study is underway to reveal the physiological roles of Ezh1 in retinal development and maintenance.

## Methods

### Mice

All animal experiments were approved by the Animal Care Committee of the Institute of Medical Science, University of Tokyo and conducted in accordance with the ARVO (Association for Research in Vision and Ophthalmology) statement for the use of animals in ophthalmic and vision research. ICR mice were obtained from Japan SLC Co. To generate retina-specific Ezh2 knockout mice, Ezh2-floxed mice^14^ were crossed with DKK3-Cre mice, which expresses the *Cre* gene in retinal progenitor cells^32^ from an early embryonic stage^14^. As controls, normal littermates mice were used. The mice used in our work is free of retinal degeneration mutations, rd1 and rd8.

### FACS sorting and cell preparation for ChIP sequence

Following number of mouse retinas was used for FACS sorting of Cd73 positive and negative population; for RNAseq, 20 (P2), 12 (P5), 20 (P8), and for ChIPseq (H3K4me3, H3K27me3), 20 (P2), 12 (P5), 24 (P8). Retinas were isolated from enucleated eyes, and incubated with 0.25% Trypsin in PBS (500 μl) for 15 minutes at 37 °C. Samples were treated with DNaseI and stained with anti-Cd73 antibody (BD Pharmingen, 550741) on ice for 30 minutes. Cells were washed and stained with propidium iodide. Cd73 positive and negative fractions were purified by using MoFlo (Beckman Coulter). Purified cells were stored at −80 °C as cell pellet until use for RNAseq. For ChIPseq, cells were resuspended in 1 ml of 2% BSA in PBS containing 27 μl of formalin. Cells were rotated at room temperature for 10 minutes, then 95 μl of 1.5M glycine was added. Cells were further rotated for 5 minutes, and washed with 2% BSA in PBS and stored at −80 °C as cell pellets.

### RNA sequencing and quantification

Total RNA was extracted from stored purified cell pellets or whole retinal cells at E15 and E18 using RNeasy Plus Micro Kit (Qiagen), and RNA quality was confirmed using a 2100 Bioanalyzer (Agilent Technologies). RNA sequence was done as previously described^33^. Briefly, the mRNA Seq libraries were constructed for each condition and conducted using the Illumina mRNA Seq Sample Preparation Kit according to the manufacturer’s instructions. Briefly, the RNA was subjected to poly(A) selection using Sera-Mag Magnetic Oligo-dT beads. Poly(A+) RNA was partially degraded by incubation in fragmentation buffer at 94 °C for 5 min. The first-strand cDNA was synthesized using random primers and SuperScript II (Invitrogen), and the second-strand cDNA was synthesized using RNaseH and DNA pol I (Illumina). Illumina GA sequencing adaptors were ligated to the cDNA ends. Double-stranded cDNA was size-fractionated by 6% PAGE, and cDNAs of 200 bp were recovered. cDNAs were amplified by 15 PCR cycles using Phusion DNA Polymerase (Finnzymes). Thirty-six-base-pair single-end-read RNA Seq tags were generated using an Illumina GA sequencer according to the standard protocol. RNA Seq tags that were mapped to the mouse reference genome sequences (mm9) without any mismatches were used. RNA Seq tags were correlated with RefSeq transcripts. All the original data set are publicized in GEO: GSE71464 (Molecular signature of Cd73 positive and negative cells in developing retina), GSE71462 (Differential expression of genes in photoreceptor and other retinal cells in developing retina), GSE71463 (Regulation of genes expression in photoreceptor and other retinal cells by histone H3 tri-methylation at K27 during retinal).

### Chromatin immunoprecipitation (ChIP) sequence

ChIP was performed as described^13^. Briefly, Frozen cell pellets (~6e + 6 cells) were resuspended in 350 μl of SDS lysis buffer (10 mM Tris-HCl pH 8.0, 150 mM NaCl, 1% SDS, 1 mM EDTA, Protease Inhibitor Cocktail; nacalai, 25955-11) and incubated at room temperature for 5 minutes. Then samples were sonicated using Sonifier 250A (Branson, output 2, duty cycle; 60% for 2 minutes) and centrifuged. For preparation of DynaBeads coupled with antibody solution, 40 μl of DynaBeads Protein G (life technologies, 10004D), 8 μg of anti-H3K4me3 antibody (Active motife, 39159) or anti-H3K27me3 antibody (Abcam, 6002) and 40 μl of SDS lysis buffer and 160 μl of ChIP dilution buffer (20 mM Tris-HCl pH 8.0, 150 mM NaCl, 1 mM EDTA, 1% Triton X-100, Protease Inhibitor Cocktail) were mixed and rotated for 4 hours at 4 °C. DynaBeads cocktail was mixed with 100 μl of sonicated chromatin solution and 400 μl of ChIP dilution buffer rotated overnight at 4 °C. Mixture was washed, and precipitates were eluted in 200 μl of elution buffer (50 mM Tris-Hcl, pH 8.0, 10 mM EDTA, 1% SDS) and incubated at 65 °C for 8 hours, then treated with Rnase A, Proeinase K, and DNA was purified using QIAquick PCR purification kit (QIAGEN). ChIP sequence was done as described previously^34^. All the original data sets are available through GEO (Fig. 1A).

### Retinal explant, and electroporation

Retinal explants and *in vitro* electroporation were performed as described elsewhere^35^,^36^. Full-length cDNAs encoding mouse Jmjd3 was obtained from Addgene (pCS2-Jmjd3-F, #17440). Amount of plasmids was 100 μ*g* for one retina. The electroporated retinas were cultured at 34 °C on a chamber filter (Millicell).

### Immunostaining

Immunostaining of sections was done as described previously^35^. The first antibodies, anti-Rhodopsin antibody (Rho4D2, kindly donated by Dr. R. S. Molday of The University of British Columbia), anti-Recoverin antibody (AB5585, Chemicon), were visualized by using appropriate Alexa Fluor dyes conjugated second antibodies (Molecular Probes). Samples were mounted in VectaShield (Vector Laboratories) and analyzed by using a Zeiss Axio Vision 4.6 microscope.

### Analysis of RNAseq Results

The sequence reads were aligned to the mouse reference genome assembly (NCBI37/mm9) using TopHat (v2.0.8b) with the option (–coverage-search –max-multihits 50)^37^. Then, fragments per kilobase of exon per million mapped fragments (FPKM) was calculated from the aligned reads by using CuffLinks (v2.1.1) with the default option^37^ as follows. The expression of each transcript was quantified as the number of reads mapping to a gene divided by the gene length in kilobases and the total number of mapped reads in millions. By using CuffLinks, 23021 genes expression values (FPKM) were estimated, and small RNAs such as miRNAs were excluded, and remaining 19954 genes were subjected to following analyses. FPKM was converted into log2 scale unless otherwise indicated.

### Analysis of ChIPseq Results

The sequence reads were aligned to the mouse reference genome assembly (NCBI37/mm9) using Bwa (v0.6.2)^38^ with default parameters. The output bam files were converted to bedgraph files using Bedtools (v2.16.2)^39^ to count the depth at each genome position. The histone modification score of H3K4me3 and H3K27me3 were calculated as follows. For test samples and input samples, the 1050 bp-window tags (50 bp sliding) were counted, and number of tags at each position was divided by the number of total tags. The values of the test samples were subtracted by that of input samples at each genome position. The values were converted into log2 scale, and the averaged values in TSS +/−5 kb (Refseq genes except for small RNAs) were subjected to quantile normalization.

### Clustering of RNAseq data by K-means and DAVID analysis, and Hierarchical clustering

Clustering of FPKM values of all the RNAseq data (Supplemental Fig. 1) was done by using K-means algorism of GeneSpring (v12.6.1, Agilent Technologies). K = 15, which gave good resolution of clustering, was used. Then the GO term enrichment analysis by DAVID (v6.7) to highlight the most relevant GO terms associated with a gene list of each clusters was performed. The p-value of GO terms with the Benjamin-value of more than 0.25 was converted into –log10. Then, we used Hierarchical clustering algorism (GeneSpring) to sub-group the 14 clusters (we omitted one cluster with almost no expression levels) after K-means based on the converted GO term p-value (Supplemental Fig. 1b). We also used Hierarchical clustering algorism (GeneSpring) to categorize bipolar-, amacrine-, and retinal ganglion-specific genes (Roska) based on the FPKM value (E15, E18 and P2, P5 and P8 samples classified into Cd73PC and Cd73NC. Then the genes, which did not show Cd73NC specific expression, were omitted (grey colored genes in Supplemental Fig. 4b–d). The remaining cell type specific gene sets (Supplemental Fig. 4e–g) were categorized again using hierarchical clustering algorism by GeneSpring based on the histone modification score of H3K4me3 and H3K27me3 (Fig. 4).

### Mapping of binding peaks of using ChIPseq data of retina specific transcription factors

For mapping of binding peaks of the transcription factor, following public available data were used; Nrl (National Eye Institute, Hong PLoS-Genet-2012)^9^, Otx2 (GSE54084)^21^, Crx (GSE20012)^20^. The sequence reads in the fastq files were aligned to the mouse reference genome assembly (NCBI37/mm9) using Bwa (v0.6.2) with default parameters. Peaks were then called on all data sets using MACS (v1.4.2). To assign target genes to each peaks we utilized the Genomic Regions Enrichment of Annotations Tool (GREAT, http://bejerano.stanford.edu/great/public/html/index.php)^40^. GREAT analysis was performed against a whole genome background and using the “basal plus extension” association rule, that defines the gene regulatory domain covering 5 kb upstream and 1 kb downstream from the TSS, plus additional regions, which correspond to extension in both directions up to 5 kb, were considered.

## Additional Information

**How to cite this article**: Ueno, K. *et al*. Transition of differential histone H3 methylation in photoreceptors and other retinal cells during retinal differentiation. *Sci. Rep.* **6**, 29264; doi: 10.1038/srep29264 (2016).

## Supplementary Material

Supplementary Information

Supplementary Information

## Figures and Tables

**Table 1:** Number and population of upregulated, or downregulated genes.

subtype	gene number	upregulated in Ezh2-CKO						downregulated in Ezh2-CKO					
		Cd73N specific (1305)		common (175)		Cd73P specific (299)		Cd73N specific (274)		common (89)		Cd73P specific (411)	
		No	%	No	%	No	%	No	%	No	%	No	%
Cd73P_A	261	21	8	2	1	18	7	10	4	0	0	2	1
Cd73P_B	36	0	0	0	0	7	19	4	11	0	0	1	3
Cd73P_C	17	2	12	0	0	5	29	0	0	0	0	1	6
Cd73P_D	60	11	18	3	5	2	3	3	5	2	3	9	15
Bipolar	61	2	3	1	2	0	0	1	2	12	20	18	30
Amacrine	76	20	26	6	8	3	4	3	4	0	0	9	12
RGC	27	6	22	4	15	1	4	2	7	0	0	0	0

Genes are considered as upregulated or downregulated only if expression is 1.5 fold higher or lower in Ezh2-CKO than control retina. Venn diagram showing overlapping of genes of upregulated or downregulated are available in Fig. 1g,h.
