# Supplementary material for: Transition of differential histone H3 methylation in photoreceptors and other retinal cells during retinal differentiation
Source: Sci Rep. 2016 Jul 5;6:29264. doi: 10.1038/srep29264 (PMC4932533; doi:10.1038/srep29264)

***Transition of differential histone H3 methylation in photoreceptors and other retinal cells during retinal differentiation***

Key words: retina, histone modification, differentiation, photoreceptors

Running title : Histone methylation pattern of retinal genes during development

Authors: Kazuko Ueno*1, 2, Toshiro Iwagawa*1, Hiroshi Kuribayashi1, Yukihiro Baba1, Hiromitsu Nakauchi3, Akira Murakami4, Masao Nagasaki2, Yutaka Suzuki5, and Sumiko Watanabe1

***Supplemental Fig. 1 Clustering analysis of RNAseq data of developing retinas using K-means***

Gene expression pattern obtained by RNAseq was clustered by using K-means into 15 groups, and the clusters were designated as C0-C14. (a) Expression patterns of each cluster. Values under cluster name are number of genes belong to each group. (b) All clusters except for C2 were served to gene ontology (GO) analysis. Then, GO terms, which have less than 0.25 Benjamini value, were chosen (Supplemental Table 1), and clustering was done using -log10 Benjamini values by DAVID analysis.

***
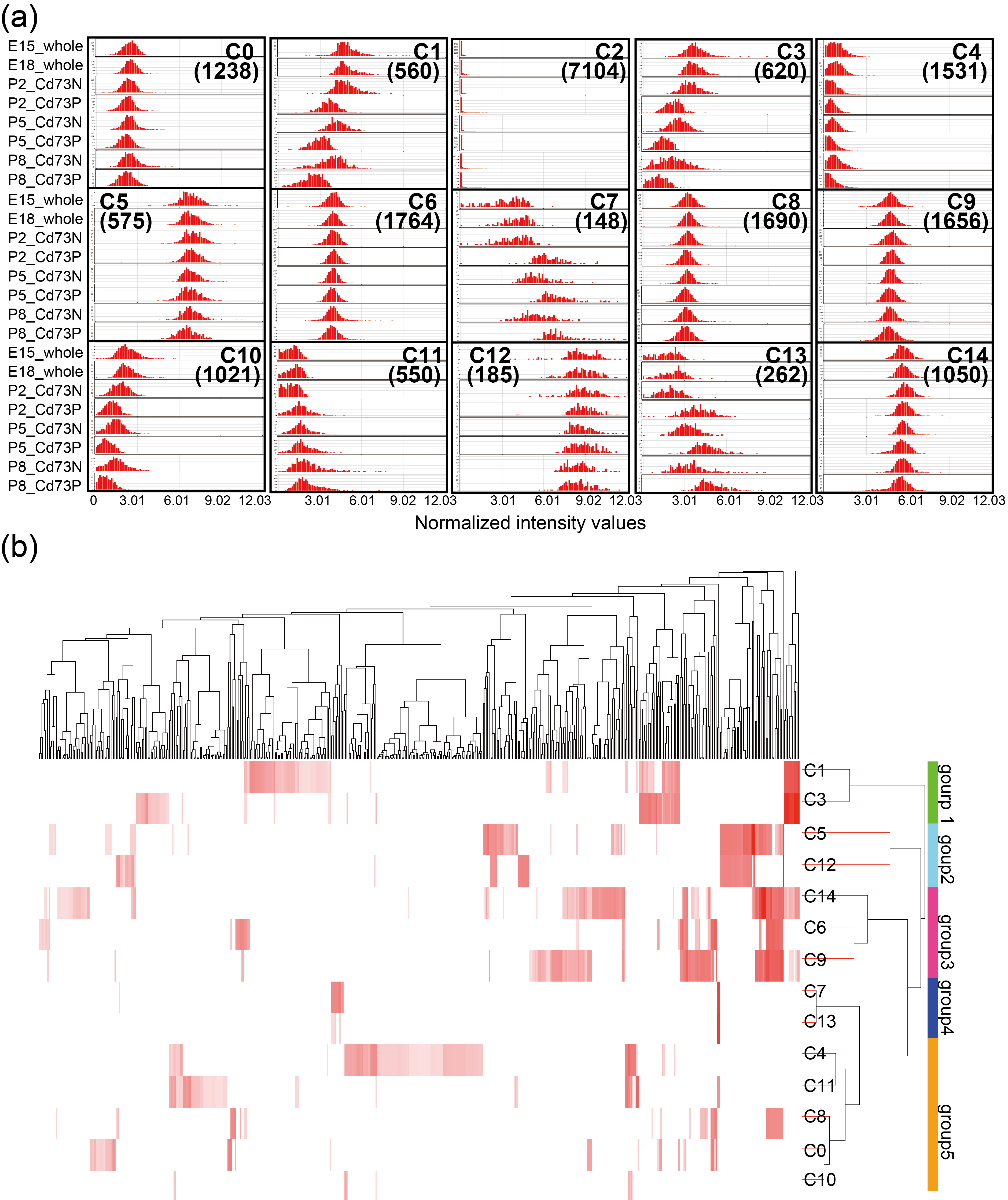
***

***Supplemental Fig. 2 Differential expression of retinal subtype specific genes in either Cd73P or Cd73N cell population, and confirmation of expression of genes with low fold change in RNAseq by qPCR***

(a) The expression patterns of several genes reported to be expressed in certain retinal cell-specific gene subsets in Cd73N or Cd73P fractions. N/P indicates % expression in Cd73NC versus Cd73 PC, and vice versa (P/N). (b-d). Expression of genes with less than 2.0-fold changes between Cd73PC and Cd73NC in the RNAseq data was examined by qPCR using newly prepared cells. Independent experiments were performed three times, and average values with standard deviation are shown. p values were calculated by student’s T test, *<0.05, **<0.01. Digits in interrupted bars indicate average and standard deviation in parentheses.

***
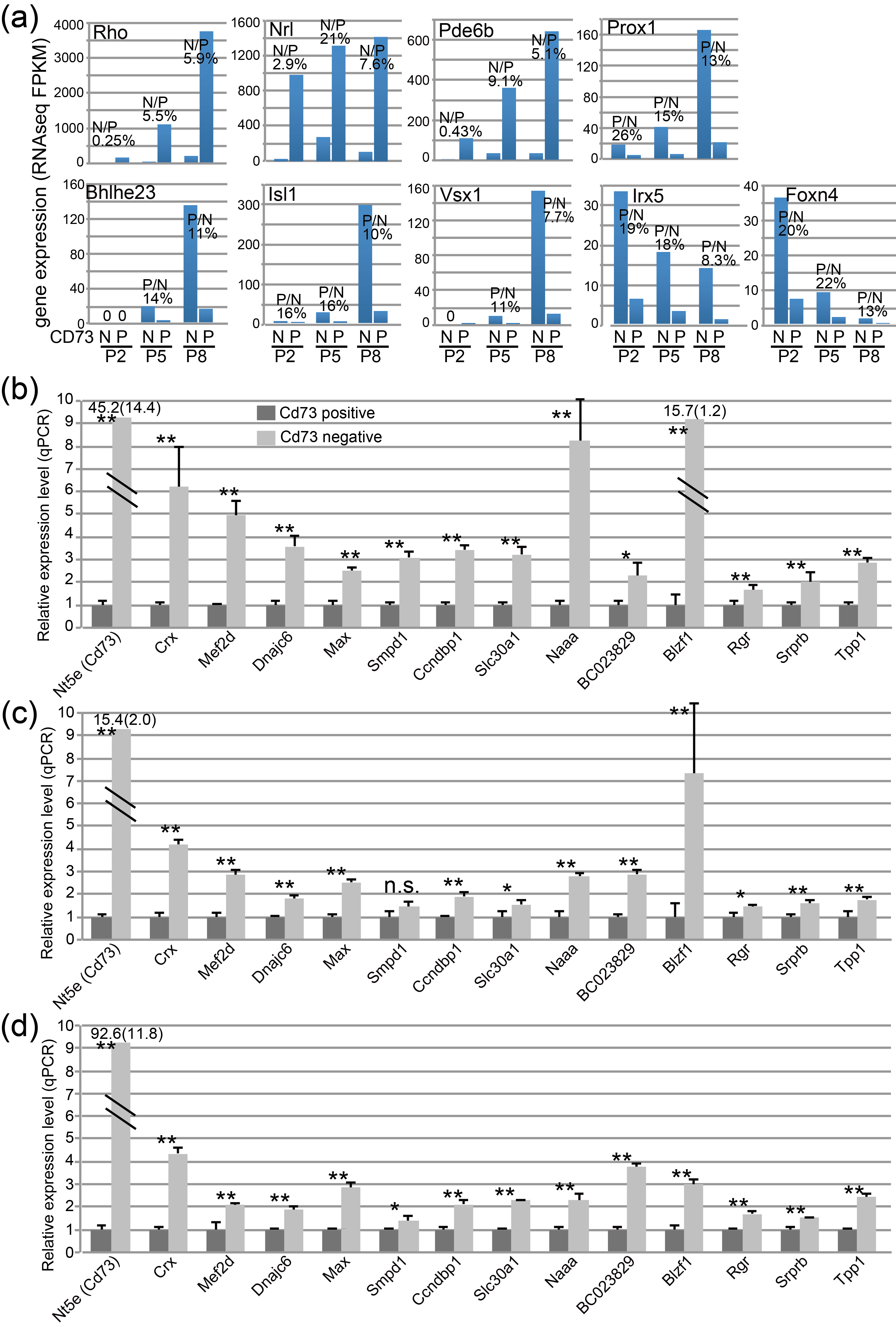
***

***Supplemental Fig. 3 Histone H3K4me3 and H3K27me3 modification of Cd73P expressing gene loci***

ChIP sequence of HistoneH3K4me3 and HistoneH3K27me3 was done using Cd73P or Cd73N retinal cell fractions at P2, P5, and P8. Cumulative values of ChIPseq signals at +/- 5 kb region of TSS of each gene was calculated. (a) Gene expression level (horizontal axis) and H3K4me3 or H3K27me3 levels (vertical axis) in cell fractions of Cd73P (Cd73PC) or Cd73N (Cd73NC) at P2, P5 and P8 are shown as scattered plot. Correlation coefficient R value is shown in the upper-right corner of each panel. Significant level alpha (two-tailed probability) was calculated by correlation coefficient r table, and red diagonal line in the panels indicates that samples have correlation value lower than 0.01. Names of genes with high values of both expression and H3 modification are shown in b and c, d, e. Genome browser tracks of H3K4me3 (d) or H3K27me3 (e) signals in gene loci of the genes in the boxes (b, c) are shown.


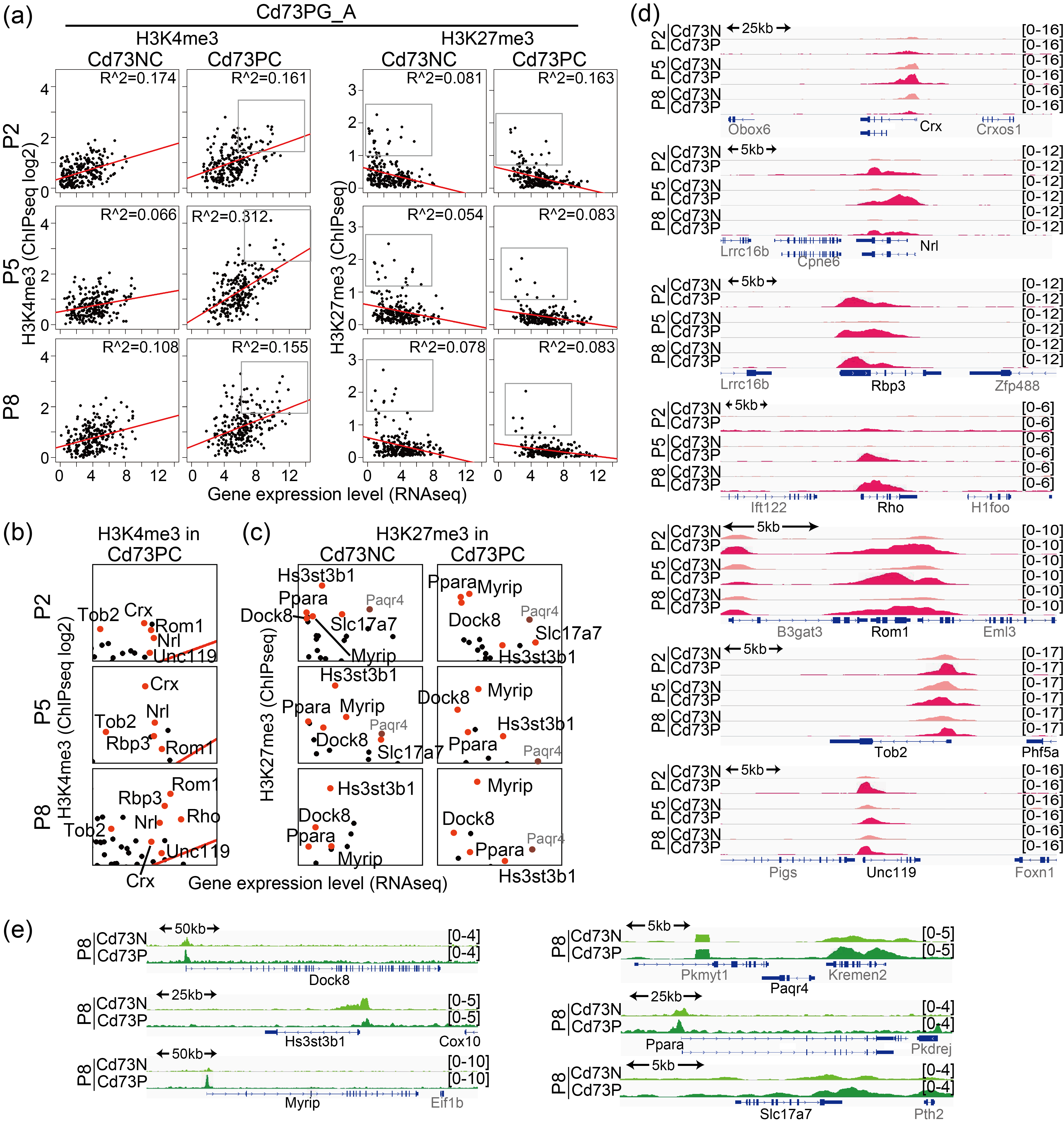


***Supplemental Fig. 4 Clustering of genes in retinal sub-population***

(a) Genes categorized to Cd73PG_A (Fig. 2) were further clustered using ChIPseq values (log2) of H3K4me3 and H3K27me3. Nine sub-clusters were designated as from C1a to C5c. B, C, D. Expression levels of genes categorized to bipolar (b), amacrine (c), and retinal ganglion cells (RGC, d) in Siegert et al. (Nat Neurosci 15, 487, 2012). RNAseq values (log2) of the genes in Cd73PC and Cd73NC fractions at P2, P5, and P8 of the genes are examined. Heat map represents relative values of gene expression in Cd73NC against CD73PC fraction. e, f, g. Amacrine, bipolar, and RGC genes selected in b-d (blue colored) are further sub-clustered by using ChIPseq values (log2) of H3K4me3 and H3K27me3. a, e, f, g. Colors in left margin indicate sub-clusters, which were designated as C1a-C5c (a), C1-C5 (e, amacrine), C1-C3 (f, bipolar), or C1-C3 (g, RGC).


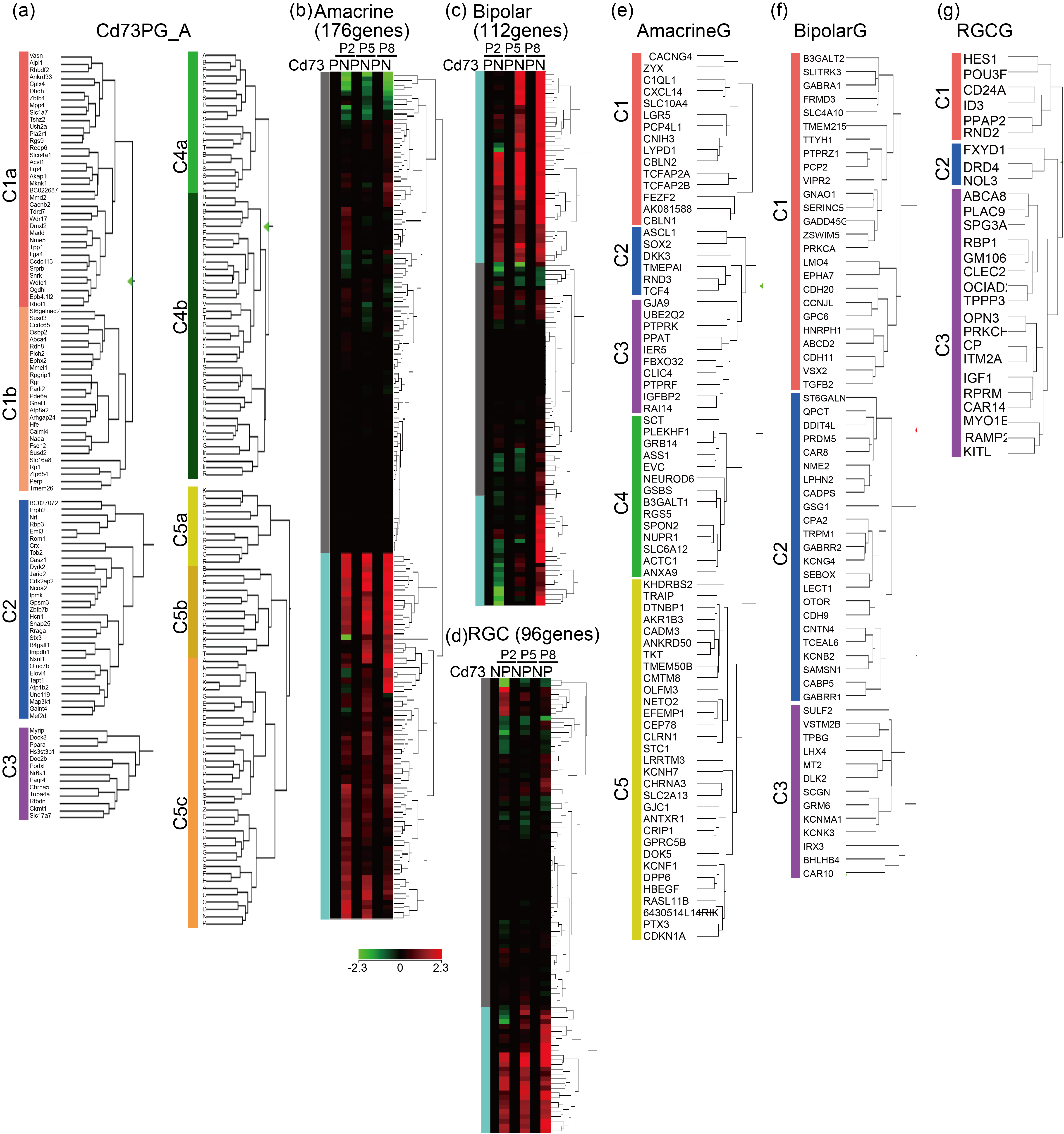


***Supplemental Fig. 5 Genome browser tracks of H3K27me3 signals of photoreceptor related genes which were upregulated in Ezh2-CKO***

(a) Genome browser tracks of H3K27me3 signals from ChIPseq data in gene loci of the Cd73P_A genes, upregulated in Cd73PC fraction of Ezh2-CKO retina. Snapshots showing the log2 ratio enrichment for H3K27me3 of Cd73NC and Cd73PC fractions at P8 wild type retina. (b) Expression level of Cdkn2a examined by RNAseq/ Gene expression levels were quantified by RNAseq data of Cd73PC and Cd73NC of control or Ezh2-CKO retina at P12. (c) Genome browser tracks of H3K27me3 signals from ChIPseq data in Cdkn2a gene locus in Cd73N and CD73PC fractions at P8 wild type retina.

***
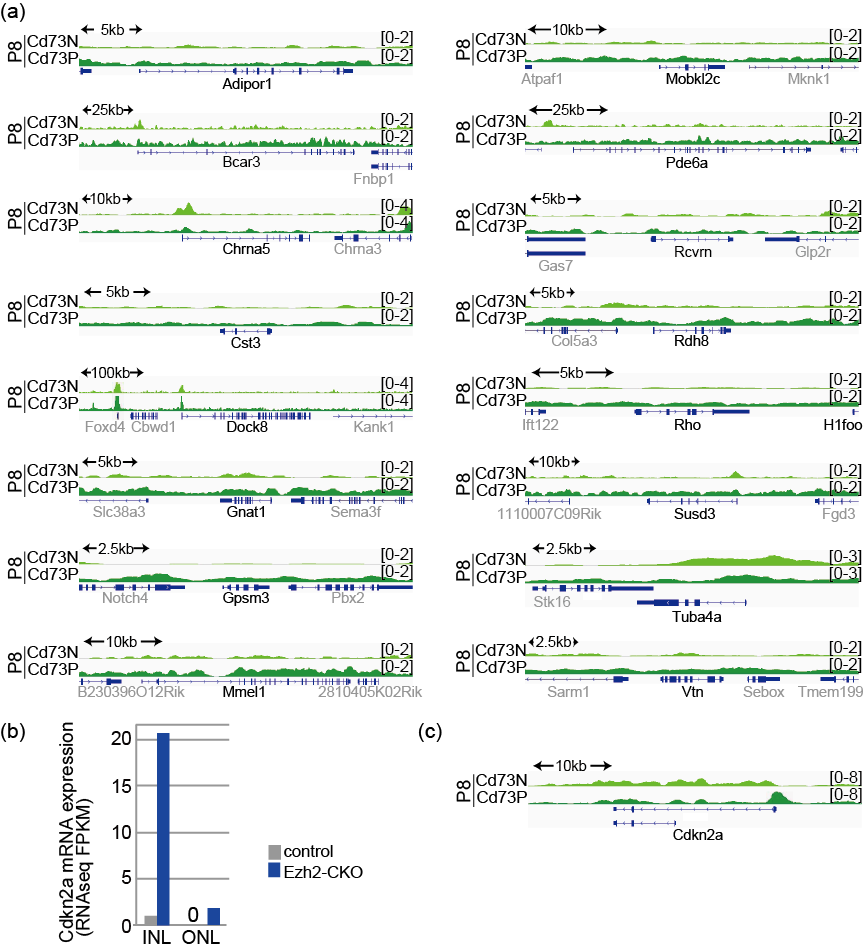
***

***Supplemental Fig. 6 Histone H3K4me3 and H3K27me3 modification of retinal genes expressed in bipolar, amacrine, and retinal ganglion cells***

List of genes specifically expressed in bipolar, amacrine, and RGC are shown in Supplemental Fig. 3. Correlation of expression level of these genes and H3K4me3 or H3K27me3 modification level (cumulative values at 5kb+/- TSS) of these gene loci is shown. Scattered plot of bipolar genes (a), amacrine genes (b), and retinal ganglion genes (c) are shown. Correlation coefficient R value is shown in the upper-right corner of each panel. Significant level alpha (two-tailed probability) was calculated by correlation coefficient r table, and red line was appeared in panels only when samples have correlation value lower than 0.05. Correlation value; ** <0.01, * <0.05.


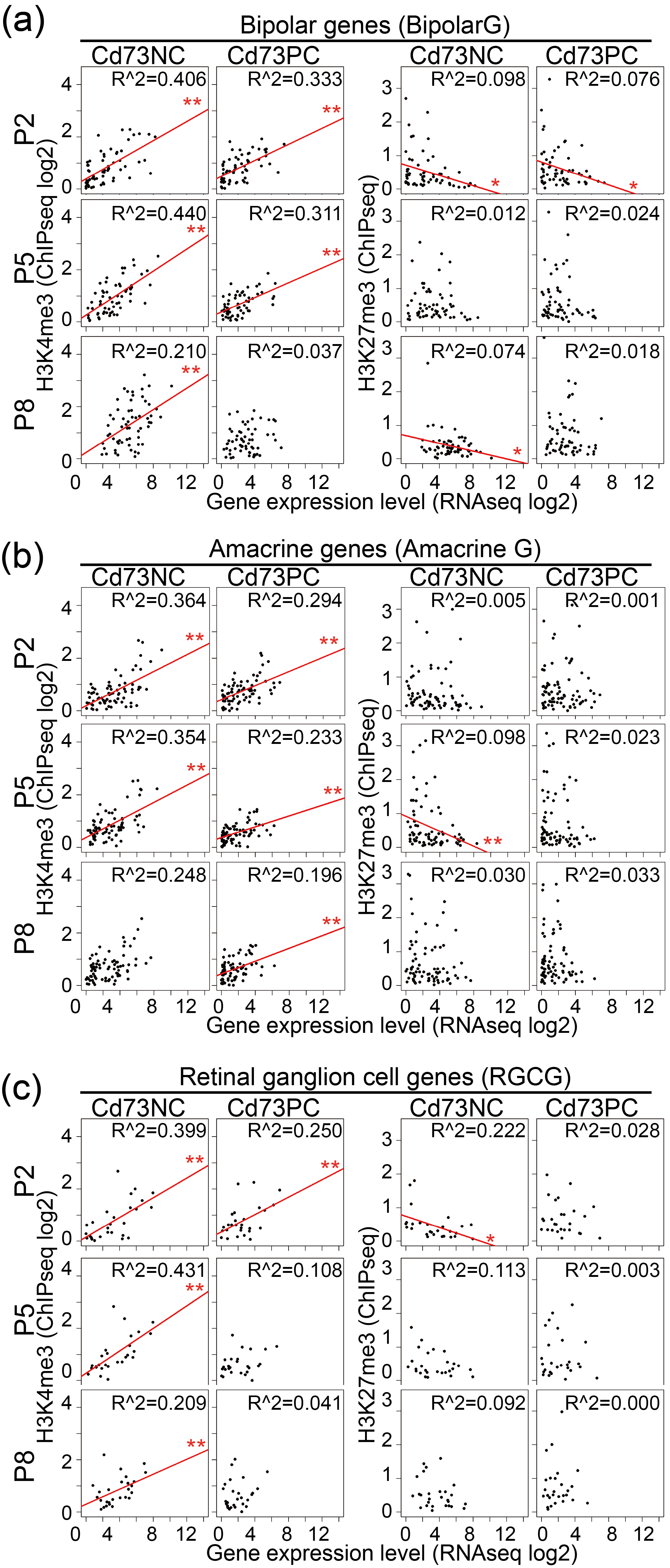

Supplement: Supplementary Information [file srep29264-s1.doc]
